# Oral Deformity

**Published:** 1885-08

**Authors:** J. A. Hooper


### THE DENTAL REGISTER.

VOL. XXXIX.] • AUGUST, 1885. [No. 8.

### COMMUNICATONS.

### Oral Deformity.

(Read before the Kentucky State Dental Society.)

DR. J. A. HOOPER.

I wish to present some facts on this subject that will show the mistakes of dentists and physicians, or any one, who attempts to correct (or advises to correct) by the extraction of the teeth. You will see why I consider this subject one of the greatest import to the dentist, and the public, as there is not a dentist in the land, I will venture to say, who is a close observer, but will endorse me when I say that from seventy-five to ninety per cent. of the present generation have irregular or deformed mouths, some to a limited, and some to a very marked extent. Would you think that in a city of one hundred thousand inhabitants, seventy-five thousand at least have deformed mouths? For the last eight or ten years I have been a close observer, and have given this subject special study and reflection, and the more I observe and the longer my mind dwells upon it the more deeply I feel that the dentist should investigate it with due deliberation, and the medical profession should be posted in regard to this subject. A short time ago a patient, in speaking of this subject in my office, informed me that he heard a professor in a medical college say, that this thing of straightening teeth was all stuff.

Last summer I heard a professor and dean of one of our leading dental colleges say in regard to oral deformities, that it was the alphabet, and should not be discussed in our socities.

In the *Ohio Journal of Dental Science* of October, 1883, Dr. W. E. Driscoll attempts to criticize a part of the address I delivered before this Society two years ago, referring to the extracting of children's teeth before the proper time, "as there is great danger of rupturing the permanent teeth;" he admits, that there are a great many extracted before the proper time, and also if I am correct there must be a great many deformed mouths; he further says, he has the first case to see yet, and winds up this paragraph by saying, "who has?" I will answer by referring him to Jno. Tomes, of London, whose book is a standard text-book in all the colleges, I believe. In his second paragraph he refers to me again as follows, "interfering with mastication, and causing constriction of the arch, producing deformity." If Dr. D. can prove that constriction of the arch results, I hope he will do so, as it is a thing often asserted, but so far, lacking a single practical illustration in my experience and observation." He says, that he is amazed that any writer can *know* the tenth part of what he asserts.

"And as we see the views expressed in very much the same language in the oldest and newest works placed before us, the belief amounts to a demonstration that much is repeated without very serious thought as to its probable consistency with actual facts, and since so many reiterate old and exploded fancies with all the solemnity of later-day revelation, it seems that one can do nearly as much good in his day and generation by assisting in such observations as in giving his time exclusively to elucidating new theories."

I think Dr. Driscoll is not familiar with what he asserts. I will show you by these casts and models of mouths I have taken. Here is an inferior maxillary; by this I will prove that Dr. D. exposed to the world, or to the readers of the *Journal*, his ignorance on this subject, and it would seem that he had never observed at all, and had never spent much thought on the subject. These casts will show what a dangerous position he (Dr. D.) and the professor and dean heretofore referred to occupy, and which if acted upon, would operate against the welfare of the public, in saying this branch of dental science is the alphabet of dentistry, and should not be discussed in our dental societies. I should judge that Dr. D. was one of his students. I think I can prove beyond a doubt, this contraction and deformity.

This cast, No. 1, is a young lady fourteen years of age, her parents consulted several dentists and it was decided to extract the first left bicuspid, which was done nine months before I took this cast. She has been deformed and maimed for life, one side of the mouth is contracted; take the line and measure and you will see that one side is nearly one-half inch shorter than the other, and the center of the mouth is drawn to the side one-quarter of an inch. You can see what deformity resulted in this young lady's mouth.

No. 2, is a young lady who has met with the same misfortune.

No. 3, is a young man from Missouri, a medical student, who is also in the same condition.

No. 4, is a gentleman about thirty-five years of age who had two teeth extracted on the left side, you can see what deformity has been produced by this, there is nearly three-quarters of an inch difference in the size of the mouth; you can see where the central part of the mouth is drawn.

No. 5, is a young man fifteen years of age, who had the first bicuspid extracted and the front teeth filed, look what deformity this has produced, the front teeth will not touch by one-quarter of an inch, so you see what filling will do.

Here is an inferior maxillary or lower jaw of an adult; you see there was a tooth extracted years before the death of this person, the opposite side is full, round, and normal, and the other side where the tooth was extracted is contracted and fallen in, and there is considerable difference in the length from the center to the angle of the jaw.

No. 6, is a cast of a young man's mouth in Washington City, he is now about twenty or twenty-two years of age. His dentist who had always had the care of his teeth, (and quite a prominent man there) said his teeth were crowded, had double rows and tusks were growing out; he extracted the so-called tusks or cuspid teeth; you will observe, gentlemen, how this young man has been disfigured and deprived of his full, round, natural arch and manly expression, and is compelled to go through life in this deformed condition ; the front of his mouth is contracted to the size of a three-year-old child, as in this cast of my daughter's mouth, who is three years of age. What fond parent would wish to see a bright boy or girl deformed in this way? What judgment would a man mete out to another who would thus disfigure his child? We should practice dentistry as we should wish it practiced in our families. There is not one case in a hundred where there is a demand for the extraction of teeth to correct irregularities, and any dentist who will extract sound teeth, because the patient wants a pretty set of false teeth, should be prosecuted for malpractice, and debarred the privilege of practicing the profession. Upon the same principle the chiropodist might say that we could dispense with some of our toes in order to have pretty-shaped feet. Here I have two casts, one before and one after treatment, this is the evidence of what can be done.

No. 1, is a young lady, an adult. I have widened the mouth from one quarter of an inch to nearly one half, and have moved the lateral incisors and one bicuspid fully one fourth of an inch. No. 2, is a young lady who was wearing a plate with an artificial tooth as the central incisor had not developed. You will perceive that the point of the tooth was just through the gum, had been in that condition for two years, in three weeks you can see what I did to a-sist nature to develop the tooth. No. 3, is a cast of a young man's mouth in Washington City, I have shown you the upper one which has been deformed, the lower right cuspid tooth extends outside of the upper tooth which would be the natural consequence, as I have shown that the upper was contracted to the size of a three year old child. You will observe that the lateral incisor had been extracted, that was done before he came to me; comparing these two casts you will see the change I made in three weeks, and he was twenty-one years of age when I did this. No. 4, is the cast of a young lady's mouth. I widened the arch and put the lateral incisor out nearly one half an inch. No. 5, is a young lady from Boston. She has been through the same treatment as there are some teeth missing.

Here is a cast six weeks after I began to treat the mouth and she only wore the apparatus at night. She says she never had any soreness or pain from it. I have had over one hundred cases and never had any that complained of soreness after the third or fourth day and but very little at any time.

Any one who undertakes this branch of dentistry without previous knowledge or study of the subject is liable to make very serious mistakes. I have complied with Dr. D's. request to prove my assertion. I have done it with actual casts of the mouth, not by pen and ink alone. There are a number of Dr. D's all over the country. There are a great many men scattered over the world practicing the profession of dentistry who have adopted the views of the professor and dean heretofore referred to, trying to hold a position against well established facts. Dr. D's position is untenable.

Gentlemen, my observation and experience compel me to differ with the old accepted theory of absorption and deposit; for instance, there are cases in which I have moved teeth from one quarter to one half an inch. The old theory is that when you move a tooth you produce absorption on the opposite side and a deposit on the side from which it was moved. If that theory were correct in the case to which I refer I certainly would have pushed the tooth through the alveoli and would have had a deposit to the thickness of the distance of the tooth moved, but from these casts you can notice scarcely any difference. My theory or experience is that if the teeth are moved rapidly enough so that the alveoli will bend in front and contract behind the tooth, as the alveoli is composed very largely of an elastic substance, and is therefore very yielding to pressure, as for instance, when an upper molar or jaw tooth is extracted the alveoli springs or yields sufficiently to get the tooth out without breaking the roots or alveoli. Suppose you take shrubbery or any young tree that is crooked and you wish to straighten it, you bend it with as rapid a pressure as possible, careful not to break or bruise it, you have not produced absorption or deposit; you have changed the form of the fibre or tissue very little, if any at all, you hold it there till it grows to the form or new position, then you leave it permanently straight, the same applies to the teeth. Further I wish to call your attention to the mistakes in our laws, which we should endeavor to correct. Men are permitted to go through the country advertising their patent medicines and claiming to extract teeth without pain, and by this indiscriminate extracting of teeth deforming thousands of people. Suppose that our surgeons through the land were thus to disfigure mankind by their surgical operations, there would not be judges and attorneys enough to attend to the suits that would be instituted against them.

